# Magnesium matters: unveiling hidden risks in kidney transplant patients through total and ionized magnesium profiling

**DOI:** 10.3389/fneph.2024.1385447

**Published:** 2024-07-16

**Authors:** Federica Bocchi, Simeon Schietzel, Uyen Huynh-Do, Bruno Vogt, Daniel Sidler

**Affiliations:** Department of Nephrology and Hypertension, Inselspital, Bern University Hospital, Bern, Switzerland

**Keywords:** magnesium, total magnesium, ionized magnesium, hypomagnesemia, kidney transplant

## Abstract

**Background:**

In kidney transplant (KT) patients, magnesium (Mg^2+^) deficiency is widespread. It is often encountered early after KT, may persist longer, and is frequently promoted by calcineurin inhibitors (CNIs) and tubular leakage. Studies demonstrated an association between post-KT hypomagnesemia and allograft dysfunction. The concentration of the active form, the ionized Mg^2+^ (iMg^2+^), is not measured clinically, and total Mg^2+^ (tMg^2+^) and iMg^2+^ correlations are conflicting. We assess the cross-sectional prevalence of hypomagnesemia in KT patients. The correlation of demographic and anthropometric parameters was also studied.

**Methods:**

A prospective, single-center analysis of KT patients was conducted at the University Hospital of Bern, Switzerland (March 2023–August 2023). Blood samples were collected at least twice for the majority of patients. tMg^2+^ has been quantified from a plasma sample at the Clinical Chemistry Department of the University Hospital of Bern. The PRIME^®^ ES analyzer (Nova Biomedical, USA) provided results for iMg^2+^. The following co-variables were considered: age, comorbidities, kidney disease, KT history, estimated glomerular filtration rate (eGFR), and treatment (including Mg^2+^ supplementation and immunosuppression).

**Results:**

A total of 208 measurements in 104 patients were performed [once in 9/104 patients (8.7%), twice in 86/104 (82.7%), and three times in 9/104 (8.7%)]. Compared to that in healthy volunteers (51 measurements in 51 participants), mean iMg^2+^ was significantly lower in KT patients {KT: 0.46 mmol/L [interquartile range (IQR): 0.40–0.50], volunteers: 0.57 mmol/L (IQR 0.54–0.61), p < 0.01}. Overall, iMg^2+^ and tMg^2+^ showed strong category agreement (r^2^ = 0.93, p < 0.01). In linear regression, low iMg^2+^ correlated with CNI exposure. For 110/208 measurements (52.9%), a reduced iMg^2+^ (cutoff: 0.42 mmol/L) was shown. In 58/208 (27.9%), both values were reduced, and 52/208 (25%) had isolated reduced iMg^2+^. In principal component analysis, patients with isolated low iMg^2+^ clustered with patients with low iMg^2+^ and tMg^2+^.

**Conclusion:**

iMg^2+^ and tMg^2+^ were strongly correlated. A substantial proportion of patients show isolated low iMg^2+^. Currently, it is unclear if these patients suffer from Mg^2+^ deficiency.

## Introduction

Magnesium (Mg^2+^) ranks as the fourth most abundant cation in the human body, playing a crucial role in various cellular and biological processes (1). Hypomagnesemia, characterized by depletion of Mg^2+^ in the blood, is mainly due to conditions that promote renal or gastrointestinal Mg^2+^ wasting and is responsible for cardiac, neurological, and neuromuscular disorders. Mg^2+^ deficit, often overlooked compared to other electrolyte disorders, raises substantial health concerns and is frequently encountered in clinical settings, affecting as much as 30% of the elderly population (2). Hypomagnesemia has been linked to cardiovascular risk and is commonly associated with unfavorable clinical outcomes and higher mortality (3–7).

Dysmagnesemia has also been linked to unfavorable outcomes in individuals undergoing dialysis and those with chronic kidney disease (CKD), acting as a potential predictor for both mortality and the deterioration of renal function in CKD patients (1, 7). In kidney transplant (KT) patients, hypomagnesemia is widespread, often encountered in the first weeks after KT, and frequently promoted by calcineurin inhibitors (CNIs), other concomitant medications (i.e., thiazide-type and loop diuretics and pump proton inhibitors), and tubular leakage (1). In more than 20% of KT patients, hypomagnesemia persists for several years after KT (6). Several studies have demonstrated that pre- and post-KT hypomagnesemia is independently associated with immune dysfunction, thus likely promoting opportunistic infections (8, 9). KT patients experiencing hypomagnesemia may also be prone to impaired glucose metabolism, increasing their susceptibility to the development of diabetes mellitus (6, 10). Moreover, associations between low Mg^2+^ levels and a more rapid decline in renal function, increased risk of graft loss (1, 6, 11), and major cardiovascular outcomes have been reported (7). This underscores the importance of paying more attention to abnormal Mg^2+^ values in clinical practice within this patient population.

Clinical laboratories typically measure total Mg^2+^ (tMg^2+^), which represents the sum of both the protein-bound (approximately 20%–30%) and the free and biologically active form of Mg^2+^ (approximately 60%–70%), also referred to as ionized magnesium (iMg^2+^). Even though protein-bound and free Mg^2+^ fractions are usually in a steady state changing in similar directions, they may also change independently from each other with complex buffering and compartment-shift mechanisms involved. Dysproteinemias may change bound Mg^2+^ fractions without necessary changes in free Mg^2+^. Acid–base disorders may affect Mg^2+^ binding affinities, resulting in altered iMg^2+^ fraction, while tMg^2+^ level remains unchanged (12, 13). Evidence suggests that tMg^2+^ may not adequately reflect a patient’s Mg^2+^ state, underestimating the prevalence of clinically relevant hypomagnesemia (14–16). Earlier studies have shown discrepancy and poor correlation between tMg^2+^ and iMg^2+^. Favoring iMg^2+^ over tMg^2+^ could enhance the detection of hypomagnesemia, enabling more accurate identification of patients in need of supplementation and mitigating the risk of toxicity (14–18). However, there is still a lack of comprehensive understanding regarding the clinical utility and the subsequent impact on therapeutic interventions. Consequently, the measurement of iMg^2+^ remains uncommon as a routine test in most clinical settings.

The study aims to prospectively investigate plasma tMg^2+^ and iMg^2+^ levels in both healthy subjects and a cohort of KT patients and to define 1) the cross-sectional prevalence of hypomagnesemia and 2) correlate this state with clinical, demographic, and anthropometric parameters.

## Materials and methods

### Study design and population

This study was conducted between March 16, 2023, and August 11, 2023, at the University Hospital of Bern, Switzerland. The study is a prospective, single-center exploratory study, designed to investigate Mg^2+^ levels in KT patients compared with a group of healthy volunteers. The study protocol was approved by the Medical Ethics Committee of the study centers (Project-ID 2022-01953), and informed consent was obtained from all the participants. Participants (KT and volunteers) were free to take part and were recruited in the environment of the primary investigator and project partners.

### Mg^2+^ measurement

tMg^2+^ was measured in venous whole-blood samples. Blood samples were collected at two instances for KT patients at least 2 weeks apart and less than 6 months apart and at one instance for volunteers’ controls. tMg^2+^ has been quantified at the Clinical Chemistry Department of the University Hospital of Bern. The PRIME^®^ ES analyzer (Nova Biomedical, Waltham, MA, USA) provided results for iMg^2+^. The tubes were not centrifuged, all analyses were performed within 6 hours of sample collection after preparation and calibration of the device, and the results were pH corrected. In volunteer participants, only iMg^2+^ was always analyzed; tMg^2+^ was analyzed on a case-by-case basis if the medical situation required a larger blood sample for another reason. iMg^2+^ concentrations were not used for clinical decision-making purposes.

### Co-variates and endpoints

KT patients’ data (including kidney disease characteristics, cardiovascular risk factors and/or disease, and medication) were collected from the clinical information platform, and laboratory analyses were extracted via the Insel Data Science Center (IDSC). The IDSC is the IT organizational unit of Insel Gruppe AG for the collection, provision, and use of digital data for scientific purposes. Investigators utilized filled questionnaires to gather medical data. A medical visit, conducted on the day of inclusion, was performed for all participants. During this visit, anthropometric measurements and blood pressure readings were collected for both groups. Additionally, a second visit, scheduled at least 2 weeks apart, was exclusively designated for KT patients.

The primary endpoint of the study was the prevalence of hypomagnesemia in KT patients, defined by repeated serum iMg^2+^ levels below reference values. Lower reference values were defined as 2 standard deviations (SDs) below mean tMg^2+^ and iMg^2+^ among volunteers, and the reference ranges were as follows: serum tMg^2+^ 0.66 mmol/L and serum iMg^2+^ 0.42 mmol/L. It is important to acknowledge that the standard reference ranges for Mg^2+^ exhibit significant variation (3%–6%) due to differences in analytical methods, as well as variations within individual subjects and between different subjects (16). Secondary endpoints include the cross-sectional prevalence of hypomagnesemia, identification of subgroups with isolated low iMg^2+^ but normal tMg^2+^, and the correlation of iMg^2+^ levels with various baseline factors [age, gender, delayed graft function (DGF), KT history, estimated glomerular filtration rate (eGFR) given in mL/min/1.73 m^2^, cardiovascular risk factors or disease, and medications].

### Exclusion criteria

Participants were excluded from the analysis if they 1) were aged ≤18 years at the inclusion, 2) were pregnant or breastfeeding, and 3) were unable to understand and sign the informed consent form. Overall, no such patients were excluded from the primary endpoint analysis.

### Patient and public involvement

Neither patients nor the public was involved in the design, conduct, or analysis of this study.

### Statistical analysis

The sample size was based on *a priori* calculations with a mean expected difference between iMg^2+^ in KT and volunteers of 0.10 mmol/L (0.45 vs. 0.55 +/− 0.15 mmol/L) and an enrollment ratio of 2:1. A minimal sample size of 78 participants (52 KT and 26 volunteers) was calculated with an alpha error of 0.05 and power of 0.8. Descriptive statistics are presented as median [interquartile range (IQR)] or as mean ± SD for continuous variables, and as numbers and percentages for categorical variables. Univariate logistic regression was performed for the dichotomous secondary endpoint of normal iMg^2+^ and low iMg^2+^ for the following dependent parameters: age, gender, KT history (years), eGFR, blood pressure, history of DGF, Mg^2+^ supplementation, CNI treatment, presence of cardiovascular risk factors [diabetes mellitus, hypertension, dyslipidemia, obesity, or obstructive sleep apnea (OSA)], and presence of cardiovascular disease (ischemic or hypertensive cardiomyopathy). Subsequently, a multivariate logistic regression analyzed normal and low iMg^2+^ for all factors listed above. Results are shown as odds ratio (OR) and confidence intervals (95% CIs). Correlation between iMg^2+^ and tMg^2+^ was established using linear regression and the strength of the correlation with Pearson’s coefficient (r^2^). Principal component analysis (PCA) was used for KT participants and was performed using the following parameters: iMg^2+^, gender, age, KT history, eGFR, Mg^2+^ supplementation, and CNI treatment. Dichotomous parameters were recoded as 0 for absent and 1 for present. Dimension 1 vs. Dimension 2 was depicted. Each point represents one patient. Color code represents patient group: normal iMg^2+^, normal tMg^2+^, isolated low iMg^2+^, low iMg^2+^, and low tMg^2+^. Statistical analyses were performed using R statistical software (version 4.0.3) and R Studio (version 1.3.1093). A two-tailed p < 0.05 was considered statistically significant.

## Results

### Selection procedure and overall characteristics of participants

A total of 208 measurements in 104 KT patients [once in 9/104 patients (8.7%), twice in 86/104 (82.7%), and three times in 9/104 (8.7%)] and 51 measurements in 51 volunteers were performed. The selection procedure is summarized in **Supplementary Figure 1**. Demographic data and clinical characteristics of participants (KT and volunteers) are shown in **Tables 1** and **2A**, **B**. The majority of KT participants were Caucasian men with an average age of 60 years (IQR 50–70). In 87% of instances, participants experienced hypertension, with dyslipidemia and diabetes affecting 41% and 30%, respectively. KT participants had undergone KT for an average of 5.1 years, with an interquartile range of 0.8 to 13.6 years. In 21% of cases, they had experienced DGF, and the mean eGFR at the study entry was 42 mL/min/1.73 m^2^. Additionally, 45% of KT participants received cyclosporine therapy and 31% tacrolimus therapy. In contrast, the majority of volunteers consisted of Caucasian women, accounting for 63% of the group, with an average age of 51 (IQR 38–59). In both groups, a small percentage of individuals were on Mg^2+^ replacement therapy—specifically, 19% in the KT group and 12% in the volunteer group.

**Table 1:** General baseline participant’s characteristics.

	KT recipients N = 104	Healthy volunteers N = 51
Age	60 (50, 70)	51 (38, 59)
Gender (female)	33 (32%)	32 (63%)
Ethnicity (Caucasian)	94 (90%)	50 (98%)
BMI (kg/m^2^)	25.2 (22.0, 28.8)	24.0 (21.4, 27.6)
BP systolic (mmHg)	138 (126, 152)	126 (120, 131)
BP diastolic (mmHg)	83 (74, 91)	82 (75, 88)
Cardiovascular risk factors		
Hypertension (yes)	90 (87%)	7 (14%)
Diabetes (yes)	31 (30%)	0 (0%)
Dyslipidemia (yes)	43 (41%)	1 (2%)
Obesity (yes)	14 (13%)	12 (24%)
OSA (yes)	3 (2.9%)	0 (0%)
Cardiovascular disease (yes)	60 (58%)	1 (2%)
Mg supplementation (yes)	20 (19%)	6 (12%)

Cardiovascular disease is defined as the presence of ischemic and/or hypertensive cardiomyopathy.

KT, kidney transplant; BMI, body mass index; BP, blood pressure; OSA, obstructive sleep apnea; Mg, magnesium.

**Table 2A:** KT recipients’ specific characteristics.

	KT recipient N = 104
DGF (yes)	22 (21%)
Transplant history (years)	5.1 (0.8, 13.6)
eGFR (mL/min)	42 (28, 55)
Kidney disease	
Glomerulonephritis	24 (23%)
Diabetic nephropathy	14 (13%)
Hypertensive nephropathy	10 (9.6%)
Genetic	
ADPKD	22 (21%)
Other	12 (12%)
Unknown/others	22 (21%)

KT, kidney transplant; DGF, delayed graft function; eGFR, estimated glomerular filtration rate; ADPKD, autosomal dominant polycystic kidney disease.

**Table 2B:** KT recipients’ therapy.

	KT recipient N = 104
Calcineurin inhibitors	79 (76%)
Cyclosporin	47 (45%)
Tacrolimus	32 (31%)
Antimetabolite	80 (77%)
Azathioprine	12 (12%)
Mycophenolate mofetil	68 (23%)
Prednisone	86 (83%)
mTor inhibitors	12 (12%)
Everolimus	10 (9.6%)
Sirolimus	2 (1.9%)
Biologicals	22 (21%)
Belatacept	22 (21%)
Tocilizumab	1 (1%)

KT, kidney transplant; mTor, mammalian Target of Rapamycin.

### Prevalence of hypomagnesemia and correlations

**Figure 1:**
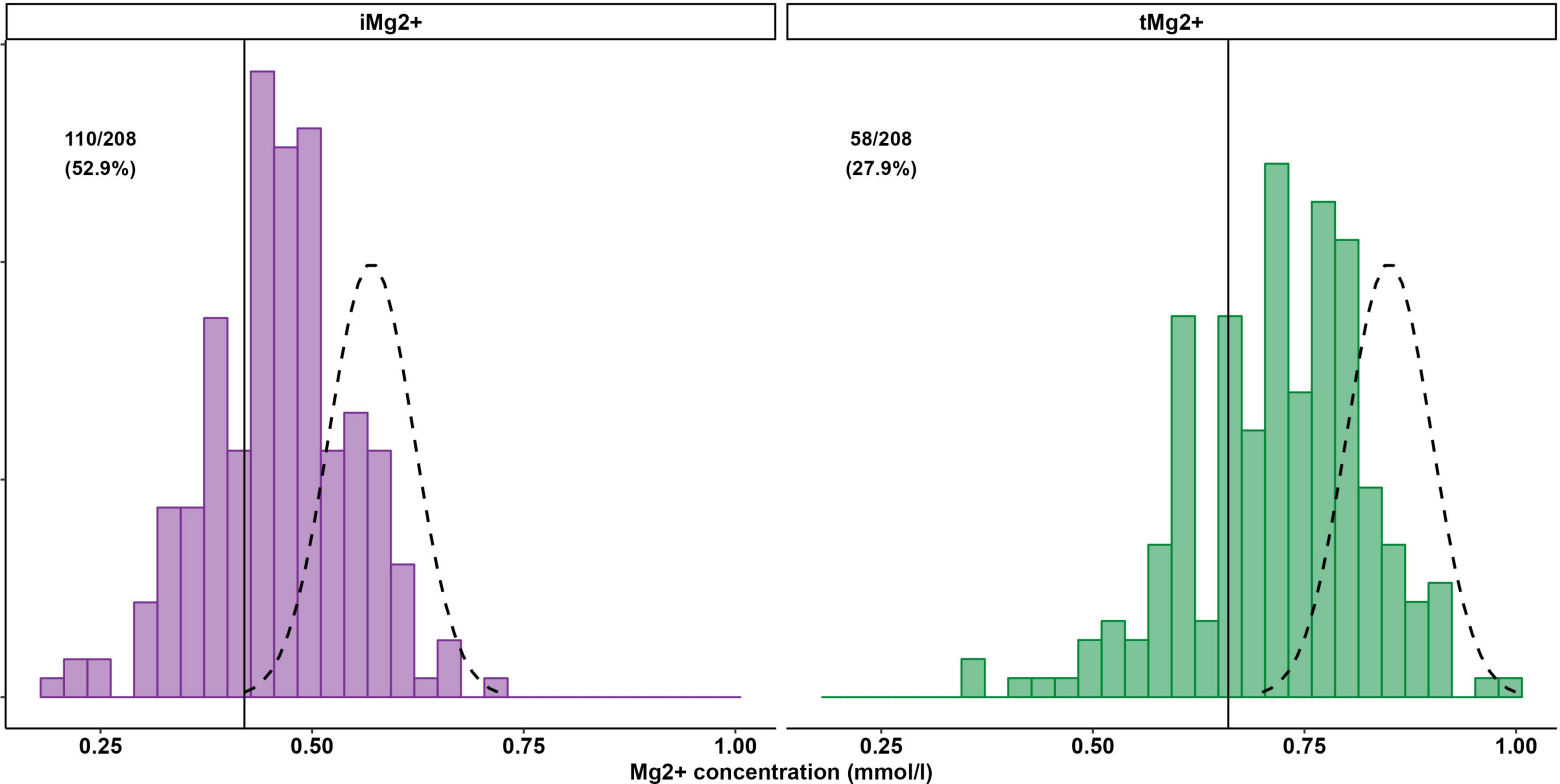
Prevalence of low iMg2+ (left side) and low tMg2+ (right side) in KT participants.

**Figure 2:**
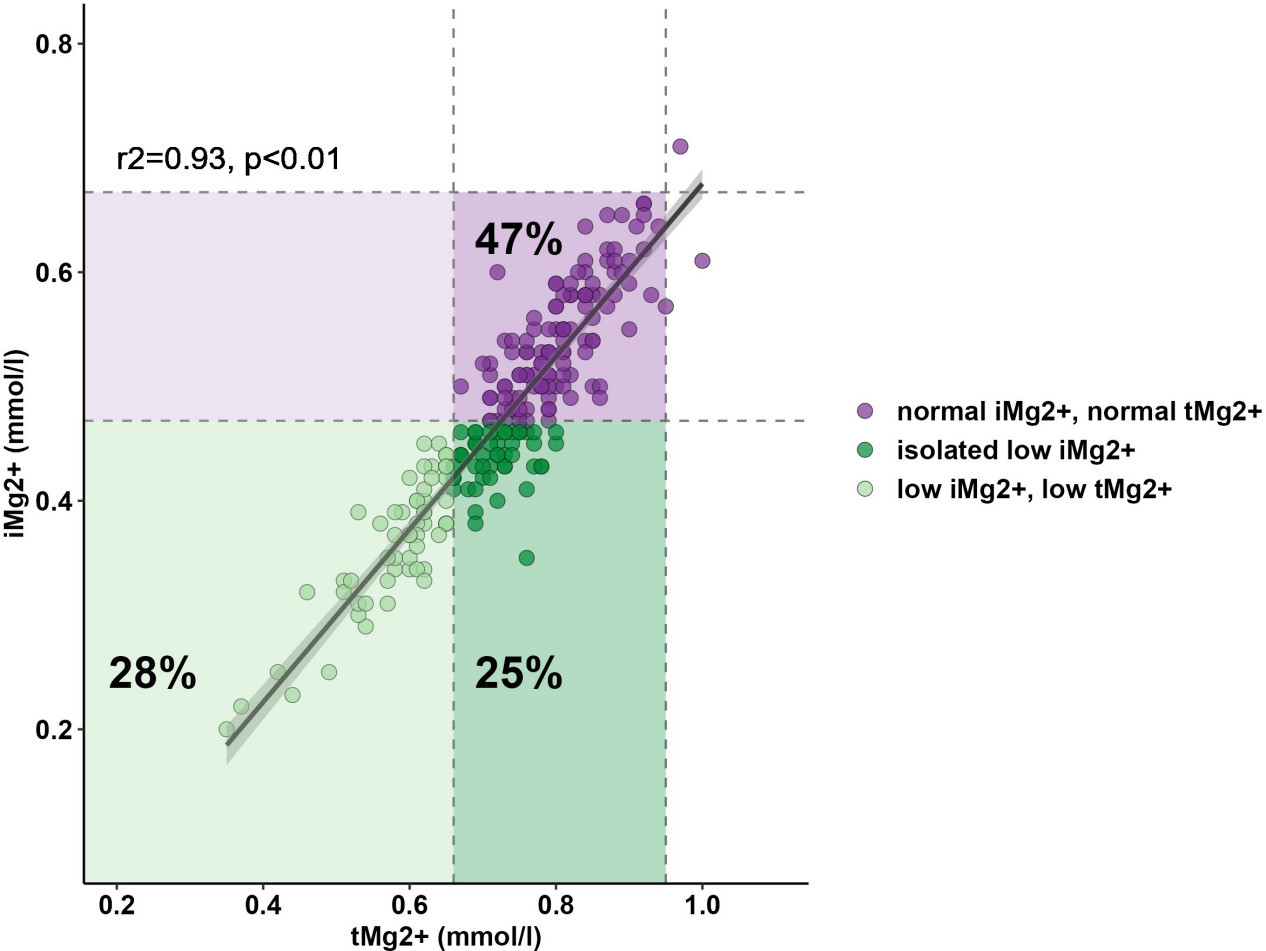
Relationship between serum tMg2+ and iMg2+. Lower reference ranges of tMg2+ (0.66 mmol/L) and iMg2+ (0.42 mmol/L) are shown and delimited with dashed lines.

**Figure 3:**
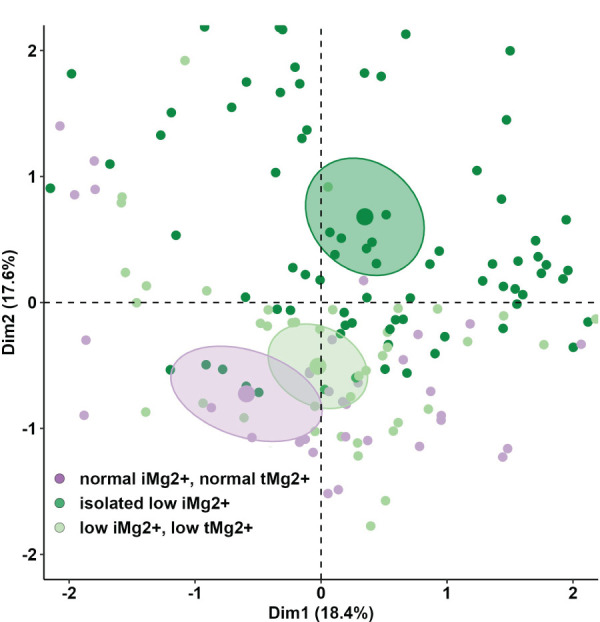
Principal component analysis (PCA) of anthropometric and demographics parameters and lab values of KT participants between the different three groups (normal iMg2+ and normal tMg2+, isolated low iMg2+, low iMg2+ and low tMg2+).

Compared to that in volunteers, mean iMg^2+^ was significantly lower in KT patients [KT: 0.46 mmol/L (IQR 0.40–0.50), volunteers: 0.57 mmol/L (IQR 0.54–0.61), p < 0.01] (**Figure 1**). For KT patients, the association between tMg^2+^ and iMg^2+^ is depicted in a scatter plot in **Figure 2**. Overall, iMg^2+^ and tMg^2+^ showed strong category agreement (r^2^ = 0.93, p < 0.01). Dashed lines delimit the reference range values for iMg^2+^ and tMg^2+^. For 110/208 measurements (52.9%), a reduced iMg^2+^ was shown. In 58/208 (27.9%), both values were reduced, and 52/208 (25%) had isolated reduced iMg^2+^. In principal component analysis, patients with isolated low iMg^2+^ clustered with patients with reduced tMg^2+^ and not with patients with normal Mg^2+^ (**Figure 3**). Considering the well-known association between CNI use and serum Mg^2+^ levels (16, 19), and the potential risks related to hypomagnesemia in KT patients, the association between Mg^2+^ values and the use of CNI was investigated. In logistic regression, low iMg^2+^ correlated with CNI exposure. No other correlating factors were identified, notably excluding factors such as eGFR, KT history, or the antecedent of prior DGF (**Table 3**).

**Table 3:** Univariate and multivariable logistic regression for the dichotomous secondary endpoint of normal iMg^2+^ and low iMg^2+^.

	Univariate			Multivariable		
	OR	95% CI	p-Value	OR	95% CI	p-Value
Characteristics						
Age	1.00	0.97, 1.03	0.9			
Gender (female)	1.94	0.84, 4.72	0.13			
DGF	1.51	0.58, 4.15	0.4			
KT history	1.02	0.97, 1.07	0.4			
eGFR	0.99	0.97, 1.01	0.3			
BP (mmHg)	1.00	0.98, 1.02	0.9			
Cardiovascular risk factors (yes)	0.39	0.06, 1.81	0.3			
Cardiovascular disease (yes)	0.79	0.36, 1.73	0.6			
Therapy						
Mg supplementation (yes)	2.12	0.77, 6.48	0.2			
Calcineurin inhibitors (yes)	4.22	1.69, 11.7	**0.003**	7.03	2.15, 26.8	**0.002**

Cardiovascular disease is defined as the presence of ischemic and/or hypertensive cardiomyopathy.

OR, odds ratio; CI, confidence interval; DGF, delayed graft function; KT, kidney transplant; eGFR, estimated glomerular filtration rate; BP, blood pressure; Mg, magnesium.

## Discussion

In this study, we investigated the add-on value of assessing serum iMg^2+^ compared to tMg^2+^ in detecting KT patients with pathological Mg^2+^ levels. The ultimate goal was to establish the cross-sectional prevalence of hypomagnesemia and examine its correlation with various clinical, demographic, and anthropometric parameters.

In a study sample including 208 measurements, we found that hypomagnesemia was frequent, with a prevalence of 52.9% (reduced iMg^2+^ and tMg^2+^). The notable high percentage contrasts with findings in other studies (20, 21), likely attributable to variations in methodology and the accepted Mg^2+^ cutoff criteria. This highlights the importance of determining the best measurement method and universal reference values. We also proved a strong correlation between iMg^2+^ and tMg^2+^ concentrations. These results have also been found in previous studies (16, 19). Most of our KT patients had CKD with moderate renal failure, and normo-magnesemia was expected. The high prevalence of hypomagnesemia in our study can also be explained by the use of CNI (76% of KT patients received cyclosporine or tacrolimus therapy). Indeed, after analysis of various factors, only the use of CNI correlated statistically significantly with hypomagnesemia. CNI induces hypomagnesemia through urinary Mg^2+^ wasting by downregulating the renal expression of the epidermal growth factor (EGF) and the Mg^2+^ channel Transient Receptor Potential Pelastatin 6 (TRMP6) in the distal collecting tubule (1, 22). The incidence of tacrolimus- or cyclosporine-induced hypomagnesemia has been studied in-depth, and these common side effects are now well known in the medical world (20, 21, 23).

Studies demonstrated that hypomagnesemia is associated with unfavorable outcomes and mortality (21, 22). Favoring iMg^2+^ over tMg^2+^ could enhance the detection of hypomagnesemia, enabling more accurate identification of patients in need of supplementation. Previous studies have investigated the sensitivity and correlations of measurements of iMg^2+^ compared to tMg^2+^ in detecting hypomagnesemia (24, 25). Our study suggests that tMg^2+^ could represent the active form of Mg^2+^ and be sufficient to detect hypomagnesemia (strong category agreement and correlation between iMg^2+^ and tMg^2+^). In contrast, the PCA illustrated that a significant proportion of patients otherwise considered normo-magnesemic on the basis of tMg^2+^ can be reclassified as hypomagnesemic if considering only iMg^2+^. This seems particularly true for patients with short KT follow-up (**Supplementary Figure 2**). The misclassification could have clinical and therapeutic implications. Non-treated hypomagnesemia is associated with poor outcomes, including allograft function and mortality (26, 27). On the contrary, the administration of Mg^2+^ supplements is not without side effects. Furthermore, there is a lack of well-established knowledge regarding the influence of substitution therapy notably, on iMg^2+^ levels, and the appropriate timing for its initiation. While some studies (28) indicate that Mg^2+^ supplementation leads to an elevation in tMg^2+^, Rooney et al. demonstrated that Mg^2+^ substitution did not produce a statistically significant alteration in iMg^2+^ concentrations (29). These inquiries merit additional investigation.

Our study also has some limitations. First, although extensive in terms of parameters evaluated, it is a prospective and single-center study. Moreover, the study sample was small, limiting the generalizability of our results. In addition, the lack of standardized iMg^2+^ reference thresholds, inter-personal variability, and relation to the device used is a further obstacle to the generalization of the results. However, the inclusion of a control group allowed us to better define the reference value of Mg^2+^ and not base it solely on the already known literature, which we further specify is variable and not well defined. Second, although correlations with drugs have been studied, only analyses with immunosuppressant and Mg^2+^ supplements have been carried out. Exploring additional variables would have been valuable, particularly investigating the usage of other commonly prescribed drugs within the KT population. This could include diuretics and pump proton inhibitors, both known to contribute to urinary Mg^2+^ losses. Additionally, concerning the use of CNI, we were unable to investigate whether there was a therapy-dependent dose effect on the occurrence of hypomagnesemia. Finally, we could not capture the untoward clinical consequences of the reported ionized hypomagnesemia, such as allograft or cardiac outcomes. The percentage of participants on Mg^2+^ replacement therapy was small, and we did not study the potential beneficial or damaging effect of supplementation.

In conclusion, the determination of iMg^2+^ as a standard of care could be useful in recognizing false normo-magnesemic patients, particularly in high-risk populations such as KT patients. Currently, it is unclear if these patients suffer from Mg^2+^ deficiency and if supplementation is indicated. Further prospective interventional trials are needed.

## Data availability statement

The original contributions presented in the study are included in the article/**Supplementary Material**. Further inquiries can be directed to the corresponding author.

## Ethics statement

The studies involving humans were approved by the Medical Ethics Committee of the study centers (Project-ID 2022-01953). The studies were conducted in accordance with the local legislation and institutional requirements. The participants provided their written informed consent to participate in this study.

## Author contributions

FB: Conceptualization, Data curation, Formal analysis, Investigation, Methodology, Project administration, Software, Supervision, Validation, Writing – original draft, Writing – review & editing. SS: Conceptualization, Investigation, Validation, Writing – review & editing. UH: Conceptualization, Investigation, Supervision, Validation, Writing – review & editing. BV: Conceptualization, Investigation, Supervision, Validation, Writing – review & editing. DS: Conceptualization, Data curation, Formal analysis, Investigation, Methodology, Project administration, Software, Supervision, Validation, Writing – review & editing.

## Supplementary material

Supplementary Figure 1 Cohort analyzed. KT : Kidney Transplant.

Supplementary Figure 2 Percentage of KT participants with normal iMg2+ and normal tMg2+, isolated low iMg2+, low iMg2+ and low tMg2+ according to KT history (0-1 year, 1-3 years and > 3 years).
