# Supplementary figures and images for: Magnesium matters: unveiling hidden risks in kidney transplant patients through total and ionized magnesium profiling
Source: Front Nephrol. 2024 Jul 16;4:1385447. doi: 10.3389/fneph.2024.1385447 (PMC11286567; doi:10.3389/fneph.2024.1385447)

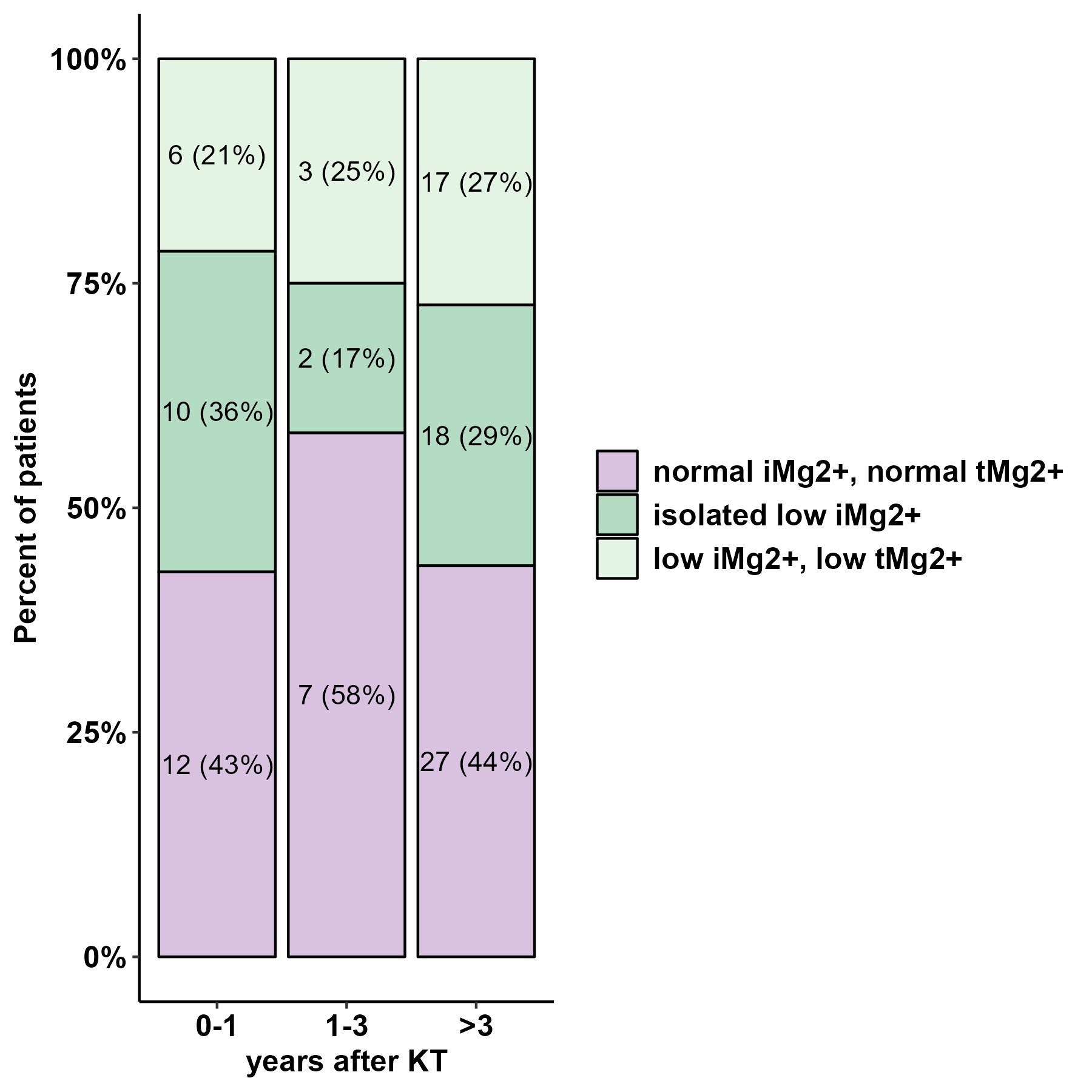

Supplement: Supplementary Figure 1 — Cohort analyzed. KT : Kidney Transplant. [file Image_1.jpeg]

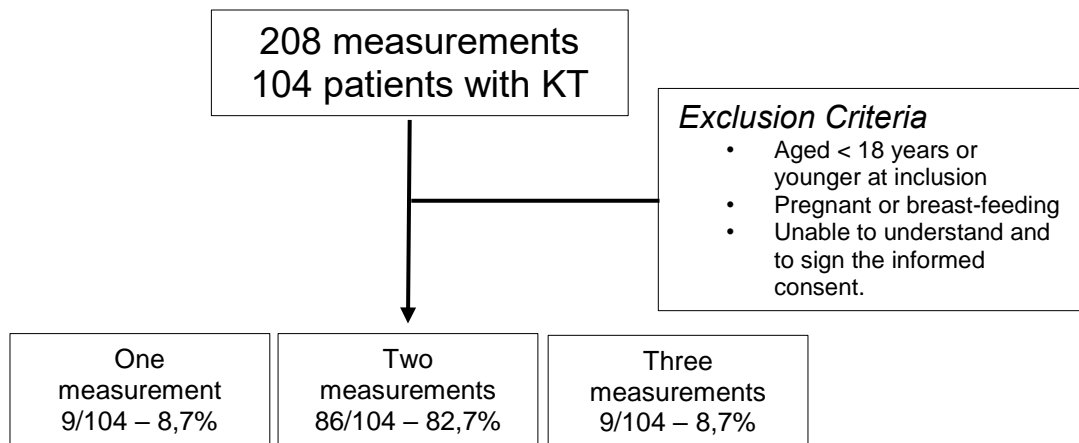

Supplement: Supplementary Figure 2 — Percentage of KT participants with normal iMg2+ and normal tMg2+, isolated low iMg2+, low iMg2+ and low tMg2+ according to KT history (0-1 year, 1-3 years and > 3 years). [file DataSheet_1.pdf]
